# Virtual reality and music's impact on psychological well-being

**DOI:** 10.3389/fresc.2022.864990

**Published:** 2022-08-11

**Authors:** Stephen Alexanian, Maxwell Foxman, Danny Pimentel

**Affiliations:** ^1^ School of Journalism and Communication, University of Oregon, Eugene, OR United States; ^2^ University of Oregon, Oregon Reality Labs, School of Journalism and Communication, Portland, OR, United States

**Keywords:** virtual reality, music therapy, mood, media, well-being

## Abstract

Quality of life is bound to psychological well-being, which in turn is affected by the frequency and magnitude of negative mood states. To regulate mood states, humans often consume media such as music and movies, with varied degrees of effectiveness. The current investigation examined the effectiveness of virtual reality (VR) vs. two-dimensional (2D) online interventions with various stimuli (audiovisual vs. visual only vs. audio only) to assess which interventions were most effective for improved well-being. Additionally, this study examined which groups displayed the highest amount of perceived presence to understand what components are essential when maximizing a person's subjective feeling of being “in” a new place and if this translated toward therapeutic results. Our data suggests that even though VR participants generally experienced more presence and had similar benefits as 2D groups for increasing positive mood, only participants in the 2D groups had a reduction in negative mood overall with 2D audiovisual participants experiencing the best results. These results contradict past studies which indicate that there could be other psychological and theoretical considerations that may play a role in determining what online experiences are more effective than just examining presence and immersive stimuli. Further research and development into using VR as a tool for improved well-being is needed to understand its efficacy in remote and in-person setting.

## Introduction

A person's psychological (subjective) well-being relates to a person's anxiety and mood levels (1–3). Research on alleviating stress and negative mood is essential since stress can trigger and influence many diseases (4). A more recent study also indicated that negative mood states can increase inflammation in the brain (5), which can lead to cancer and tumor progression (6). In an increasingly stressful environment heightened by the COVID-19 pandemic, which has seen 40% of people reporting at least one adverse mental condition (7), it is essential to find solutions to help people's psychological well-being.

This study seeks to examine a possible promising method of enhancing well-being for the general public by combining online environments (two-dimensional and virtual reality) with music. Both VR (8) and music (9) have been used in the past to improve people's well-being. However, there has been limited research conducted using VR and music together to understand if there are any additional potential health benefits for improving people's well-being.

Music used in therapeutic ways (Music therapy) “is the clinical and evidence-based use of music interventions to accomplish individualized goals within a therapeutic relationship by a credentialed professional who has completed an approved music therapy program” (10). Music used in this study was not guided by a licensed music therapist, but participants followed the receptive music therapy model in which participants are asked to listen and respond to recorded music (11). Additionally, the music selected was based on criteria from other music therapy studies (12–14) that include music based on (1) steady pulse, (2) quiet mood, (3) predictable melodic lines, (4) little dynamic change, (5) supportive bass line, (6) stability in volume and (7) low pitches to ensure the quality was similar to that of a music therapy session. Music is not only able to stimulate the brain area (basal ganglia) that regulates feeling of reward (15), but also can decrease the release of cortisol (a stress hormone) such as when a person is attending a musical performance (16). Furthermore, several pilot and experimental studies focused on positive aspects of music when relieving stress for patients such as those who are terminally ill (17, 18) and indicating that music alone can reduce stress and arousal (9) by lowering blood pressure (19).

Research has indicated that listening to “soothing and calming music” is the type of audio most beneficial for reducing stress compared to listening to nature sounds such as rippling water (20), p. 4. Additional research has concluded that classical music and designer music (music that is designed to have an effect such as Doc Lew Childre's composition of Heart Zones) showed the most promising results for relaxation and mental clarity (21) because those genres are more efficient in stimulating the sympathetic nerve (22). Other research focused on the effectiveness of preferred music of people versus non- preferred music and concluded that while preferred genres and artists by people are significantly more effective at reducing pain (23), preferred music was not significantly more stress-relieving than non-preferred music (24).

In addition to audio's noted impact on mental health, visual media, particularly virtual reality (VR), also exerts influence over users' psychological well-being as a modality. Virtual reality is associated with head-mounted displays (HMDs), which are used in tandem with three-dimensional (3D) or 360-degree video virtual environments (25). While there are differences in defining this technology, the clinical and cognitive viewpoint of virtual reality mainly provides a subjective experience that makes the user believe the experience is real and he/she is there (26).

VR research has indicated that the immersive environments can create strong feelings of “presence for participants” (27, 28). In addition, Lombard and Ditton (29) have studied the various components of presence, which includes (1) realism, (2) transportation, (3) immersion, and (4) media richness. Realism can be characterized to the extent of which the virtual environment emulates the real world (30) and requires high costs for improving quality environments, which can be challenging. Furthermore, researchers examined three different types of transportation that relate to spatial presence such as: (1) when a user is transported to another place, (2) an object or place moves toward a user and (3) two or more users are moved to a certain place (29).

The present study focuses on the first type of transportation (i.e., self-transportation). While some may mistakenly define “presence” and “immersion” being the same thing; immersion is an objective level of sensory engagement provided by VR whereas presence is a subjective sense of a user being “in” a new place (31). While immersion and presence are different in terms of definition, they are closely tied together and if immersion is significantly high, higher levels of presence will also be reported (32). Additionally, another researcher's study indicated that people who are more cooperative and self-transcendent may experience more presence indicating how personality may play a role in how a person interacts in immersive environments (33).

VR is particularly promising in the context of mental health because of the platform's media richness, or the number of sensory inputs provided by the platform (e.g., audio, visual, haptic). The Media Richness Theory indicates that communication outcomes depend on a variety of cues that help the receiver resolve uncertainty (34) and some forms (e.g., modalities) are more effective in conveying information (35). Cable and Yu (36) further explored modalities compared to media richness in their study finding that certain technologies have certain qualities that allow for the possibility of higher levels of media richness such as electronic bulletin boards compared to websites. Furthermore, it is generally regarded that visual input dominates the other senses, but auditory perception is also equally important. As Larsson et al (37) states: the “temporal natural of sound” leads to the view that “something is happening” – even if visuals aren't present (p. 5). Hruby (38) reported the positive impact of sound on a user's level of “being there” in a review of virtual cartography.

VR continues to develop in terms of increased presence due to substantial improvements in technology including: (1) computer graphics, (2) processing power and (3) head mounted displays (25), which results in higher levels of interactivity between the environment and person (39, 40). VR has been increasingly studied in a diverse amount of online environments (41, 42) as a health relief for people, even though it is a relatively new technique in the field (43). A 2017 review of VR in medical health settings indicated that its use has been relatively safe and resulted in patient satisfaction (44). When researchers are exploring VR relaxation and stress reduction, they either focus on using “generic environments” in which participants are passive users or environments that require users to be active by interacting with the VR place to train emotion (45). Further studies have indicated that VR provides beneficial health outcomes such as lowering symptoms of depression, relieving stress, and increasing positive mood states (46–48). A newer study confirmed the positive capabilities of VR in counteracting anxiety while drawing blood, increasing happiness and calmness and reducing negative mood states like tension and fatigue (49).

While there are exciting possibilities with music in VR environments such as live concerts (50) and people indicating high satisfaction in those environments that have music (51, 52), there has been limited research within this area for enhancing well-being. Past research combining VR with music has focused on using nature sounds having a positive effect on people in virtual environments (41), increasing enjoyment from exercising (53), and better learning outcomes (54). Research that used VR and music found success in rehabilitation of memory-related cognitive processes (55) and anxiety with phobias such as heights (56). There is limited research when it relates to examining positive mood, but one study examined how music and VR can help patients during chemotherapy improve their mood states and alleviate anxiety. Furthermore, there has been a lack of research as it relates to enhancing well-being for the general population using VR and music compared to specifically using it for clinical populations (i.e. people with depression, cancer, and PTSD).

Therefore, in light of the presented gap in knowledge concerning music combined with VR online environments, the following research questions are proposed:

**RQ_1_**: Are the effects of music stimuli on mood different across VR and 2D modalities?
**HP_1_**: Individuals that are in VR environments across audiovisual conditions are likely to experience a different total mood than those who are in 2D audiovisual conditions?
**RQ_2_**: What are the independent and combined effects of audio and visual stimuli on total mood across VR and 2D modalities?
**HP_2_**: Individuals who experience (1) virtual reality and (2) experience more media richness (i.e., both visuals and audio) are more likely to report better perceived well-being than those who experience (1) 2D and (2) less media richness (i.e., only visuals or audio).
**RQ_3_**: What are the independent and combined effects of audio and visual stimuli on on people's perceived presence across VR and 2D modalities?
**HP_3_**: Individuals who experience (1) virtual reality and (2) experience more media richness (i.e., both visuals and audio) are more likely to report higher perceived presence than those who experience (1) 2D and (2) less media richness (i.e., only visuals or audio).

## Materials and methods

### Design

To understand how delivering music through VR may contribute to someone's mental well-being, the current study employed a 2 (modality: VR vs. 2D) x3 (media richness: audio only vs. visual only vs. audiovisual) between-subjects experimental design. The study used a pretest and posttest with participants experiencing either one of the 2D groups (2D audiovisual, 2D visual only, or 2D audio only) or VR groups (VR audiovisual, VR visual only, or VR audio only). While there was no control group in this study, participants were randomly assigned to one of six conditions based on questions assessing their certain criteria, such as their access/use of VR equipment. Informed Consent and University Institutional Review Board approval was obtained before collection.

### Participation and recruitment

Participants were recruited *via* convenience sampling between April 4—April 10 (2021) and were older than 18 years of age. They were recruited online from Amazon Mechanical Turk (online crowdsourcing website) due to restricted effects from recruiting and conducting the study in-person because of the current covid-19 pandemic in 2021. Participants received $2.50 by completing the study. Ultimately, we recruited 90 participants (56 males, 33 females, 1 prefer not to say) internationally. Full participant demographics are available in Table 1.

**Table 1:** Demographics of participants.

Variables	2D Groups (*n* = 45)	%	VR groups (*n* = 45)	%	Total	%
Gender						
Male	27	60	29	64	56	62.2
Female	17	38	16	36	33	36.7
Prefer not to say	1	2	0	0	1	1.1
Age						
18–24	5	11	2	4	7	7.8
25–34	21	47	25	56	46	51.1
35–44	9	20	12	27	21	23.3
45–55	5	11	5	11	10	11.1
55–64	3	6	1	2	4	4.4
65+	2	4	0	0	2	2.2
Race						
Asian	16	36	4	9	22	22.2
African American	2	4	12	27	14	15.6
White	24	53	27	60	51	56.7
Native Hawaiian	0	0	1	2	1	1.1
Biracial	3	7	1	2	4	4.4

We did not formally screen participants for mental or physical health ailments. However, we did screen participants’ susceptibility to simulation-sickness, which is defined as characterized when there is a conflict between visual motion information and the VR scene that causes blurred vision and vertigo (57). Any participant who answered “somewhat likely” to “very likely” to the simulation-sickness question item concerning experiencing simulation-sickness was excluded from the VR groups. Additionally, to attain valid responses, participants were also excluded if they failed the attention check questions.

### Online experience footage

Participants in the 2D groups were exposed to calming visualizations *via* a 2D screen. Participants in the VR groups were exposed to the same visualizations *via* an HMD, immersing users in a 360-degree environment that created spatial sound around them. Across these conditions, participants were also randomly assigned to one of three versions of the stimuli: audiovisual, audio only, and visual only. Participants in the audiovisual condition were exposed to both visual and auditory stimuli. Participants in the audio only were exposed to only the auditory sounds (music). Participants in the visual only were exposed to the visualization without audio accompaniment.

**Figure 1:**
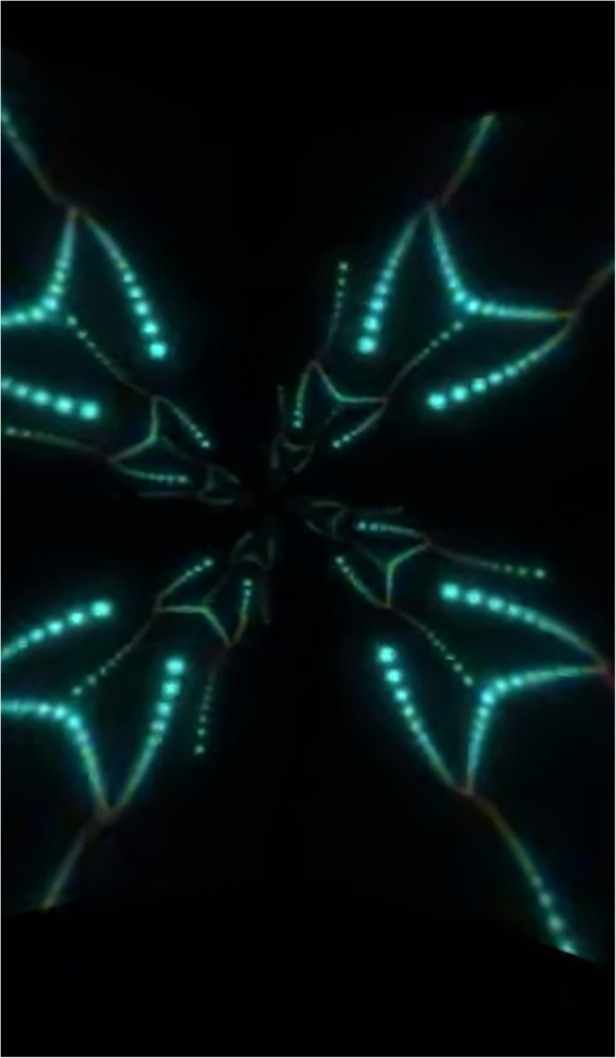
Frame from the 2D experience.

**Figure 2:**
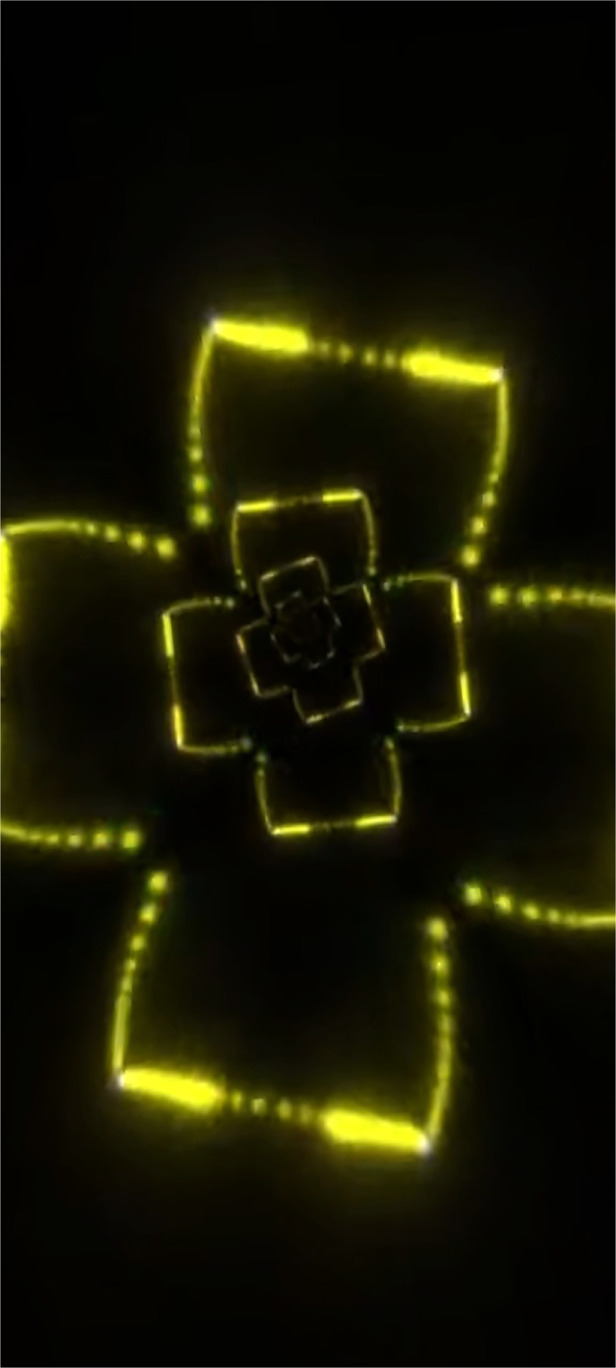
Frame from the 360-degree VR experience.

The six groups consisted of a choice of music based on empirical studies and theories of music that are associated with slow tempo and low pitches as being beneficial for reducing anxiety (12). The footage for this interaction was originally taken from a YouTube video named “Nocturnes by Candlelight - Deep Sleep and Relaxation”, which included the instrumental song, Piano at Midnight, composed by Andrew Holdworth (58). The full video on YouTube was imported into Adobe Premiere Pro to create a video lasting 3 min and 13 s long while also creating the three environments (audiovisual vs. visual only vs. audio only) that varied in sensory richness. Additionally, this video was selected due to the specific color shades and movement to fit with beats of the song in order to achieve the emotional purpose of the song (relaxation). The 2D experience consisted of only these elements. Conversely, The VR experience was created from using the above approach while additionally using Adobe Premiere Pro to create visuals to be on every side as the participant moves their head around in a 360-degree video. Both Figures 1 and 2 show visuals that participants would experience in both 2D and VR conditions but those in the VR (visual only and audiovisual) would be able to see visuals in all directions. Furthermore, as the participant moves their head around in the YouTube 360–degree environment, they experience spatial audio, but the researchers won't collect information regarding participants head movements due to not being able to have access to their VR devices.

### Device used to access survey

The one-item question collected the participants' device that was used to access the study on Amazon Mechanical Turk to assess their eligibility for continuing the study. Participants who did not select “laptop” or “desktop” were dropped from the study due to the study needing a bigger screen to fully view the experience in 2D and understand the questions asked of them.

### VR inclusion/exclusion assessment questionnaire

Due to this study being conducted online *via* Amazon Mechanical Turk, participants had to own their own VR devices to be included in the VR treatment. To assess if participants were eligible to be in one of the three VR conditions (audiovisual, visual only, or audio only), participants answered up to three questions regarding owning their own VR headset. If participants met all three conditions, they were randomly placed into one of the three VR conditions. Participants were asked if they currently own a VR headset and if they indicated “Yes,” they continued to the next VR question. The next one-item question was collected as a means of identifying any group of potential VR simulation sickness symptoms (e.g., nausea and dizziness) that could cause a negative reaction for the participant. The item was assessed on a 4-point Likert scale and any participant who selected “somewhat likely” or “very likely” to experience simulation-sickness was randomly assigned to one of the 2D groups. Conversely, any participant who selected “somewhat unlikely” or “very unlikely” was randomly assigned to one of the VR groups (audiovisual, visual only, or audio only). Additionally, the last question related to what VR device they owned and if they selected “Other,” they were randomly assigned to one of the 2D conditions instead of continuing to the VR experience due to the YouTube VR App only being compatible with certain VR devices.

### Mood

To evaluate participant's perceived mood and psychological distress, all participants completed the Abbreviated Profile of Mood States Questionnaire (POMS) before and after the online 2D or VR experience. The POMS (59) consists of seven mood dimensions but one dimension, confusion, was dropped from the study due to past studies indicating that it was less reliable (60). In addition, this questionnaire was shortened due to concerns with the reliability of data collected through Amazon Mechanical Turk (61) to reduce measurement bias (62), p. 129. The abbreviated POMS consists of a 5-point Likert scale with 35-items that evaluates the current emotional mood state of participants in terms of their level of tension, anger, fatigue, depression, esteem-related affect, and vigor. The total mood distance (TMD) is calculated by [tension + depression + anger + fatigue] - [vigor + esteem-related affect]. Overall, higher scores for TMD and negative mood states indicate higher negative mood while higher scores for positive subscales indicate better mood outcomes for participants. Reliability coefficients (Cronbach's alpha) scores for pre-test and post-test POMS subscales ranged from tension as (*α* = 0.90) and (*α* = 0.92), anger as (*α* = 0.93) and (*α* = 0.93), fatigue as (*α* = 0.91) and (*α* = 0.92), depression as (*α* = 0.93) and (*α* = 0.95), vigor as (*α* = 0.79) and (*α* = 0.82), and esteem-effect as (*α* = 0.65) and (*α* = 0.75).

### Spatial presence

To evaluate participants' perceived sense of being in the virtual space (spatial presence), participants completed the Spatial Presence Experience Scale (SPEC) after completing the experience and post-test POMS. The SPEC (63) consists of 8-items on a 5-point Likert scale. The Spatial Presence Experience Scale was calculated by taking the average of the 8-items. Higher scores for the participants indicate higher perceived presence. The reliability coefficient (Cronbach alpha) for the scale was very reliable (*α* = .93).

### Procedure

The experiment lasted approximately between 15 and 25 min, with all participants completing the study individually without the researchers interfering with the process other than to provide written instructions that were on Qualtrics. Participants could take as long as they needed to read through the instructions but there were time minimums for each page and attention check questions to make sure they understood the instructions as to ensure quality of what was being measured. Participants provided online written informed consent upon entering the study from Amazon Mechanical Turk. After agreeing to the informed consent form, participants completed the demographic and inclusion/exclusion questions, the latter dictating the random assignment of participants to the 2D or VR conditions.

Participants in the VR conditions completed additional questions to determine how much experience they had in immersive virtual environments. After completing the initial stage questions, participants completed the abbreviated POMS scale (pre-test) and then were evenly randomly assigned to one of the following: (1) 2D groups (audiovisual, visual only, or audio only) or (2) VR groups (audiovisual, visual only, or audio only). Participants in the 2D conditions completed the experience after reading instructions on how to launch the content on their browsers. Participants in the VR conditions were also given detailed instructions on how to successfully access the content through the YouTube VR App on their personal virtual reality devices, after which they watched and/or listened to the experience in their headsets. Additional instructions ensured participants across all groups removed/minimized any toolbars and accessed the content in “full-screen mode”.

After the experience was completed, participants completed the online experience attention check question. Participants who passed the attention check were then directed to the remaining survey items, consisting of the abbreviated POMS scale (post-test) and the short-form SPEC. After participants completed the post-test questions, all participants filled out three additional questions concerning their experience. Participants were also afforded the opportunity to comment on the experience and pose any questions/concerns.

## Results

### Data analysis

All of the analyses for the three research questions were performed using SPSS 27 application (IBM Corporation, Somers, New York, USA). Additionally, all participants who completed the pre-test and post-test were included in the analysis study. Participants were asked how clearly they were able to see and/or the experience and 87 participants said that the experience was either “extremely clear” or “somewhat clear” indicating that the data could be used for the study though 5/15 participants in the VR visual only group indicated that some visuals seemed to be “strobing”.

Individual participant scores for the pre-test and post-test were averaged and differences in means were compared to create the mood change variables used for the analysis. To assess differences in mood across the experimental groups, a one-way ANOVA was conducted. The results demonstrated that there were no significant differences across Time1(pre-test) Mood Disturbance, [*F*(5, 84)=1.66, *p* = .15].

To address RQ1, a two-way repeated-measures analysis (ANOVA) was applied to compare the pre-test and post-test group mean scores (mood change) between the six (3 VR vs. 3 2D) groups from the psychological test questionnaires (POMS).

To address RQ2, a series of pairwise comparisons using Fisher's LSD test were conducted from the same data used from the two-way repeated analysis (ANOVAs) to examine differences in mood disturbance over time across the experimental conditions. Furthermore, analysis using post-hoc pairwise comparisons using Fisher's LSD test for all groups were conducted to further understand what experimental condition was needed for effective improved well-being and if any conditions were more effective than others when reducing total mood disturbance.

Additional analyses using post-hoc pairwise comparisons using LSD test were conducted to understand which (if any) intervention(s) were more effective when reducing negative mood subscales (i.e., anger, tension, depression, and fatigue) and increasing positive mood subscales (i.e., vigor and esteem-related effect) compared to other groups. Higher means scores for mood change indicated a worse mood change while lower scores in the post-test indicated that the intervention was effective.

To address RQ3, a two-way analysis of variance (ANOVA) was used to assess if there were any significant differences across online interventions and a person's perceived presence. Further analyses using *post hoc* pairwise comparisons using Fisher's LSD tests were also conducted to further illustrate which groups experienced the most presence.

### Differences in VR and 2D environments

Changes in mood from pre- to post-test were examined across the six conditions. Overall, the results of the two-way repeated measures (ANOVA) showed a significant difference in interaction effect between online experiences and total mood disturbance (*p* = 0.026). Additionally, the results indicated a main effect in mood change over time across online environments (*p* < .001). Results are reported in Table 2.

**Table 2:** Univariate Effects for the two-way ANOVA analysis.

Dependent Variable	Effects	DF	F	*p* Value	*n_p_* ^2^
Total Mood Disturbance	Change*	1	45.290	<0.001	0.350
	Mood change* Online experience*	1	2.705	0.026	0.139

Computed using alpha = 0.05.

*Significant.

### Media richness and modality differences across environments

The study then investigated whether (modality: VR vs. 2D) and/or (media richness: audio only vs. visual only vs. audiovisual) influenced the magnitude of mood change among online interventions across time (mood change) to understand which online interventions were effective. A series of pairwise comparisons were conducted and the results indicated a main effect of modality on mood disturbance such that participants in every 2D condition experienced a significant decrease in mood disturbance, whereas there were no significant changes in the VR conditions as is illustrated in Table 3.

**Table 3:** Descriptive statistics for total mood disturbance levels by POMS.

Intervention	Time	Mean	SD	LL	UL	*P* value	*n_p_* ^2^
2D Audiovisual*	pre-test	2.758	4.137	0.664	4.852	<0.001	0.248
	post-test	0.013	4.070	−2.047	2.073		
2D Visual only*	pre-test	0.789	3.426	−7.746	9.324	<0.001	0.147
	post-test	−1.197	3.885	−3.163	0.769		
2D Audio only*	pre-test	0.138	5.187	−2.487	2.763	0.002	0.107
	post-test	−1.518	5.384	−4.243	1.207		
VR Audiovisual	pre-test	3.004	4.627	0.663	5.436	0.101	0.032
	post-test	2.139	4.669	−0.224	4.502		
VR Visual only	pre-test	3.778	2.727	2.398	5.158	0.441	0.007
	post-test	3.374	2.236	2.242	4.506		
VR Audio only	pre-test	2.616	4.743	0.216	5.016	0.072	0.038
	post-test	1.664	4.828	−0.779	4.107		

Computed using alpha = 0.05.

*Significant.

**Figure 3:**
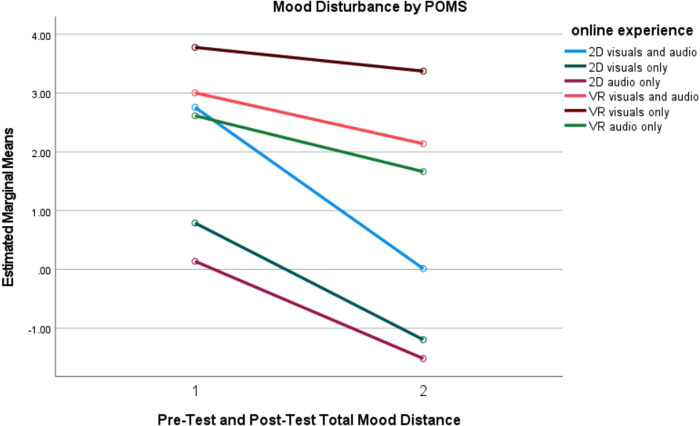
Mood disturbance measured by POMS for online experiences.

Moreover, pairwise comparisons indicated that there was significantly less mood disturbance for participants in the 2D visuals only group than those in the VR visuals only group (*p* = 0.014). Lastly, the 2D audio only participants experienced significantly less mood disturbance than participants in the VR audiovisuals group (*p* = 0.034) and the VR visual only group (*p* = 0.006). There were no other significant differences between any of the other groups. Figure 3 reports the mood disturbance estimated means for each of the online experiences between pre-test and post-test.

### Mood subscale results

Additionally, a series of repeated measures analysis (ANOVAs) with the levels of the six mood states, as measured by POMS (i.e., tension, depression, anger, fatigue, vigor and esteem-related affect) and online experience were conducted using the ANOVA test. Results of the univariate effects from the analysis of covariance (ANOVAs) are reported in Table 4.

**Table 4:** Univariate effects for analysis of covariance (ANCOVA).

Effect	Dependent variable	DF	F	*p* Value	*n_p_* ^2^
Mood change	Tension*	1	4.121	0.046	0.244
	Depression*	1	22.711	<0.001	0.213
	Anger	1	1.424	0.236	0.017
	Fatigue	1	1.688	0.197	0.146
	Vigor	1	0.605	0.439	0.007
	Esteem-related Affect*	1	219.636	<0.001	0.076
Mood change* Online experience	Tension*	5	5.411	<0.001	0.244
	Depression	5	1.742	0.134	0.094
	Anger	5	2.056	0.079	0.109
	Fatigue*	5	2.863	0.020	0.146
	Vigor	5	0.236	0.946	0.014
	Esteem-related Affect	5	1.390	0.236	0.076

Computed using alpha = 0.05.

*Significant.

The results from the two-way repeated measures analysis (ANOVAs) of the subscales indicated that an interaction between the time (mood change) and across online experiences was only significantly different for both tension and fatigue subscales (*p* < .05). Conversely, there was a significant main effect of online experiences for participant's mood change for tension (*p* = 0.046), depression (*p* < .001), and esteem-related affect (*p* < .001).

**Figure 4:**
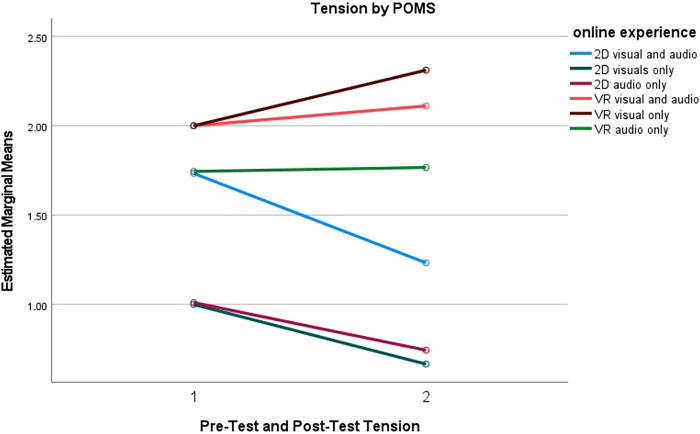
Tension measured by POMS for online experience.

**Figure 5:**
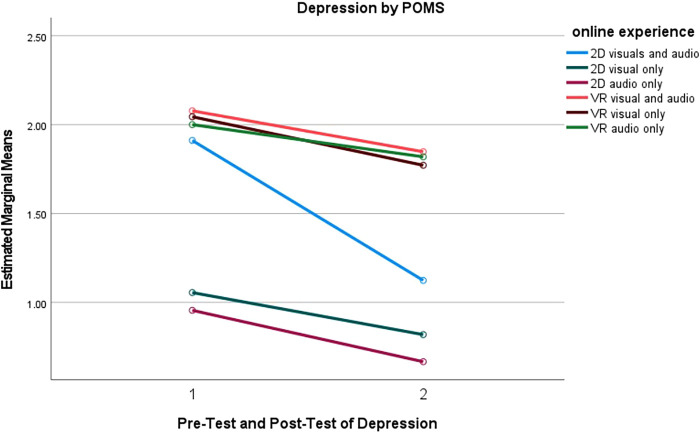
Depression measured by POMS for online experience.

**Figure 6:**
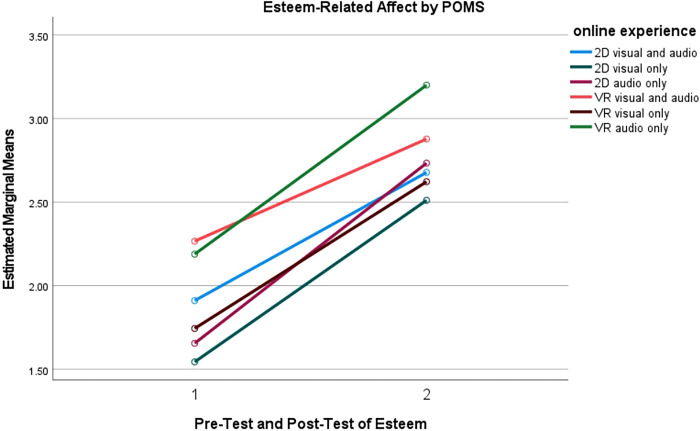
Self-esteem affect measured by POMS for online experience.

Furthermore, a series pairwise comparisons were conducted for tension, depression and esteem-related affect subgroups to understand what online experiences were more effective than others. The mean differences between online experiences are reported in Figures 4–6. Full results for both positive and negative subscales are reported in Table 5.

**Table 5:** Descriptive statistics for the Profile of Mood States negative subscales, for each group by the TIME factor, and significance differences from pairwise comparisons.

Measure	Intervention	Time	Mean	SD	95% CI		*p* value	*n_p_* ^2^
					LL	UL		
Tension	2D Audiovisuals*	pre-test	1.733	1.083	1.185	2.282	<0.001	0.146
		post-test	1.233	1.010	0.722	1.744		
	2D Visuals only*	pre-test	1.000	0.992	0.498	0.602	0.013	0.071
		post-test	0.667	0.951	0.185	1.148		
	2D Audio only*	pre-test	1.011	1.209	0.399	1.623	0.046	0.046
		post-test	0.744	1.134	0.170	1.318		
	VR Audiovisuals	pre-test	2.000	1.148	1.426	2.574	0.402	0.008
		post-test	2.111	0.742	1.736	2.486		
	VR Visuals only*	pre-test	2.000	0.779	1.606	2.394	0.021	0.062
		post-test	2.311	0.742	1.935	2.687		
	VR Audio only	pre-test	1.744	1.130	1.172	2.316	0.867	0.000
		post-test	1.767	1.190	1.165	2.369		
Anger	2D Audiovisuals*	pre-test	1.744	1.278	1.097	2.391	<0.008	0.082
		post-test	1.267	1.218	0.651	1.883		
	2D Visuals only	pre-test	1.011	1.188	0.410	1.612	0.102	0.031
		post-test	0.722	1.085	0.173	1.271		
	2D Audio only	pre-test	0.733	1.109	0.172	1.294	0.527	0.005
		post-test	0.722	1.341	0.043	1.401		
	VR Audiovisuals	pre-test	1.833	1.208	1.222	2.444	0.949	0.000
		post-test	1.844	1.181	1.246	2.442		
	VR Visuals only	pre-test	1.878	0.825	1.461	2.295	0.800	0.001
		post-test	1.833	0.611	1.524	2.142		
	VR Audio only	pre-test	1.700	1.075	1.156	2.444	0.312	0.012
		post-test	1.878	1.193	1.274	2.482		
Fatigue	2D Audiovisuals*	pre-test	1.533	1.036	1.009	2.057	0.019	0.064
		post-test	1.213	1.021	0.697	1.730		
	2D Visuals only	pre-test	0.907	0.928	0.437	1.377	0.236	0.017
		post-test	0.747	1.024	0.229	1.265		
	2D Audio only	pre-test	0.933	1.102	0.375	1.491	0.139	0.026
		post-test	0.733	1.081	0.186	1.280		
	VR Audiovisuals	pre-test	1.987	1.175	1.392	2.582	0.373	0.009
		post-test	1.867	1.158	1.281	2.453		
	VR Visuals only*	pre-test	1.853	0.863	−434,900	436.700	0.019	0.064
		post-test	2.173	0.919	1.708	2.638		
	VR Audio only	pre-test	1.947	1.215	1.332	2.562	0.692	0.002
		post-test	2.000	1.290	1.347	2.653		
Depression	2D Audiovisuals*	pre-test	1.911	1.352	1.227	2.595	<0.001	0.201
		post-test	1.124	1.144	0.545	1.703		
	2D Visuals only	pre-test	1.056	1.080	0.477	1.635	0.170	0.022
		post-test	0.819	1.143	0.241	1.397		
	2D Audio only	pre-test	0.956	1.391	0.352	1.560	0.095	0.033
		post-test	0.667	1.151	0.084	1.249		
	VR Audiovisuals	pre-test	2.078	1.536	1.301	2.855	0.182	0.021
		post-test	1.848	1.293	1.194	2.502		
	VR Visuals only	pre-test	2.044	0.878	1.600	2.488	0.114	0.029
		post-test	1.771	0.887	2.322	3.200		
	VR Audio only	pre-test	2.000	1.252	1.366	2.634	0.293	0.013
		post-test	1.819	1.090	1.267	2.371		
Esteem-related Affect	2D Audiovisuals*	pre-test	1.911	0.684	1.565	2.257	<0.001	0.246
		post-test	2.678	0.683	2.332	3.024		
	2D Visuals only*	pre-test	1.544	0.853	1.112	1.976	<0.001	0.342
		post-test	2.511	0.689	2.162	2.860		
	2D Audio only*	pre-test	1.656	0.790	1.256	2.056	<0.001	0.392
		post-test	2.733	0.881	2.287	3.179		
	VR Audiovisuals*	pre-test	2.267	0.666	1.930	2.604	<0.001	0.172
		post-test	2.878	0.717	2.541	3.215		
	VR Visuals only*	pre-test	1.744	0.462	1.510	1.978	<0.001	0.300
		post-test	2.878	0.619	1.336	4.420		
	VR Audio only*	pre-test	2.189	0.450	1.068	3.310	<0.001	0.362
		post-test	2.622	0.520	1.327	3.917		
Vigor	2D Audiovisuals	pre-test	2.253	0.996	1.749	2.757	0.490	0.006
		post-test	2.147	0.927	1.678	2.616		
	2D Visuals only	pre-test	1.640	1.085	1.091	2.189	1.000	0.000
		post-test	1.640	1.020	1.124	2.156		
	2D Audio only	pre-test	1.840	0.923	1.373	2.307	0.666	0.002
		post-test	1.773	1.178	1.117	2.369		
	VR Audiovisuals	pre-test	2.627	0.855	2.194	3.060	0.863	0.000
		post-test	2.653	0.890	2.203	3.103		
	VR Visuals only	pre-test	2.253	0.648	1.924	2.581	0.302	0.013
		post-test	2.093	0.751	1.713	2.473		
	VR Audio only	pre-test	2.587	0.648	2.259	2.915	0.931	0.000
		post-test	2.600	0.524	2.335	2.865		

Computed using alpha = 0.05.

*Significant.

#### Tension

Pairwise comparisons indicated significant decreases in tension levels between pretest and the posttest for every 2D group. Conversely, while caution should be used when indicating any clear significance due to a small effect size, tension significantly increased for the participants in the VR visual only group. Full results can be found in Table 5. Moreover, pairwise comparisons indicated that participants in the 2D visuals only group experienced less tension than participants in the VR audiovisual (*p* = 0.012) and VR visuals only (*p* = 0.012) groups. Figure 4 reports the tension estimated means for each of the online experiences between pre-test and post-test.

#### Depression

The results from the POMS scores for depression indicated that the sample (*N* = 90) used was not above the cutoff of >7 for clinical depression. Individuals were asymptomatic so caution should be used when indicating any significance for clinically depressed populations. Pairwise comparisons indicated significant reductions in the depression levels between the pretest and the posttest for only the participants in the 2D audiovisuals group as illustrated in Table 5. Moreover, pairwise comparisons indicated that participants in the 2D visuals only group had significantly less depression levels than those in the participants in the VR audiovisual (*p* = 0.016), VR visual only (*p* = 0.022), and VR audio only (*p* = 0.022) groups. Additionally, participants in the 2D audio only online experienced significantly less depression level than those in the VR audiovisuals (*p* = 0.007), VR visuals only (*p* = 0.010), and VR audio only (*p* = 0.010). There were no other significant interventions or differences among the groups for depression. Figure 5 reports the depression estimated means for each of the online experiences between pre-test and post-test.

#### Esteem-related affect

Pairwise comparisons showed significant increases in esteem-related affect levels between the pretest and the posttest for every VR and 2D group in Table 5. Pairwise comparisons indicated that participants in the VR audio only group experienced more esteem-related affect than participants in the 2D visuals only (*p* = 0.002), 2D audio only (*p* = 0.013), and VR visuals only (*p* = 0.037) groups. Additionally, participants in the VR visuals only group experienced more esteem-related affect than participants in the 2D visuals only (*p* = 0.008) and 2D audio only (*p* = 0.043) groups. There were no significant differences between any of the conditions for esteem-related affect. Figure 6 reports the esteem-related affect estimated means for each of the online experiences between pre-test and post-test.

### Online interventions and perceived presence

**Figure 7:**
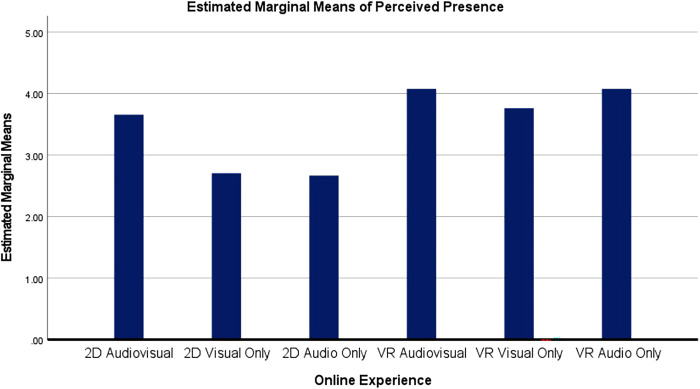
Graph of estimated mean differences for perceived presence by online experience.

In order to assess if any of the online intervention groups had a significant impact on someone's perceived presence, a two-way analyses of variance (ANOVA) was conducted and the results indicated that there were significant differences across the various online intervention groups and someone's perceived presence [*F*(5, 84)=7.482, *p* < 0.001, *n_p_*^2^ = 0.308)]. Moreover, pairwise comparison showed participants in the VR audiovisuals and VR audio only groups experienced more perceived presence than participants in the 2D visual only and 2D audio only groups (*p* < 0.001). Participants in the VR visual only group experienced more perceived presence than participants in the 2D visual only and 2D audio only group (*p* = 0.002). Additionally, participants in the 2D audiovisuals group experienced more perceived presence than participants in the 2D visuals only group (*p* = 0.005) and the 2D audio only (*p* = 0.004). See Figure 7 for estimated mean differences for perceived presence by online experience.

## Discussion

In this study, participants were randomly placed in various online environments to understand if there were any differences between the experimental conditions and mood. Overall, the results from the various statistical analyses indicated that broadly speaking, there were significant differences across the 2D and VR groups, which indicated that modality and media richness may play a role in people's music experience (RQ1).

Furthermore, additional analysis also showed that 2D environments were the only conditions to significantly reduce total mood disturbance (TMD). Examining the results more closely showed significant benefits for the participants who were in the 2D audiovisual group as that condition led to every negative mood subscale significantly decreasing over time. 2D audio only and 2D visuals only conditions both were beneficial in reducing tension. Conversely, no VR group significantly reduced any negative mood states. In fact, the VR visual only group was the worst online treatment since participants had adverse effects in which their tension and fatigue levels increased. However, as reported in Table 5, the effect size was very small so one should use caution when concluding that VR has negative consequences on people's well-being in general.

Despite these counterintuitive findings for reducing negative mood, participants in every 2D and VR groups significantly increased in esteem-related affect (positive mood). Furthermore, as reported in Table 5 the effect sizes for these results were the largest of the results from this study and VR groups had significantly better esteem-related affect than the 2D audio and visual groups. These findings indicate that modality and media richness do play a significant role into what online environment is effective but runs counter to the prior assumption that these conditions would make VR environments more effective for both positive and negative mood (RQ2).

While the results from RQ_2_ might predict that the 2D groups would experience the greatest amount of presence, the results indicated that VR groups experienced more presence than every 2D group other than 2D audiovisual. These results generally align with past research indicating that people in VR would experience the most presence, but these results also suggest that if participants are in a sensory rich environment (2D audiovisual), it may not be as essential for people to only experience online environments meant to enhancing well-being in VR (RQ3).

### Theoretical and applied implications

The results from the analyses support past literature showing promising results for music (21). However, these findings partly contradict past research presented in the literature indicating that VR would be a very effective tool for improved well-being (46, 48) and deserves discussion. Additionally, the findings appear to mostly suggest that presence may not play as significant a role in determining what experience is most effective treatment since 2D groups were overall significantly more effective.

Furthermore, discussion around presence should not solely focus on immersion of various visual and audio stimuli but also explore how users can interact more within their online environment. Additionally, using VR has mostly been studied in specific research populations, such as chemo patients, burn wound victims, and people with depression (47, 64, 65), indicating that there should be serious inquiry into how VR can be applied to the general public and be effective.

These results also suggest that there are psychological challenges when implementing VR online that are not prevalent for 2D experimental conditions. Instructions for participants for VR conditions were much more strenuous than the 2D participants due to the onboarding process of downloading the YouTube app and selecting the right settings for the experience. While the instructions were not intentionally designed to be confusing, it was still a more tedious process, which could have presented a cognitive overload (i.e. when a person's memory is overloaded due to limited working and short-term capacity) for the participant (66). While taking this theory under consideration, it makes sense that VR groups would increase in fatigue, which would then impact how people experience the environment and would degrade performance, attention, and other mood states (67). This also relates to appraisal of emotion theory in which participants may have based their emotions off their experience with the set-up resulting in a negative appraisal of response (68) causing unintended consequences.

Participants were also allowed to leave comment(s) at the end of the survey with additional feedback and 5/15 participants in the VR visual only group specifically indicated they found the visuals to have a “strobing” effect. Based on the results and some of the participants' comments, participants could have become photosensitive to the stronger visuals within VR compared to the 2D conditions. A former study also found that people diagnosed with Parkinson's disease started developing visual hallucinations that were not included in the VR environment (69) which further brings up the question if VR is only for a subset of people that are not photosensitive or have cognitive impairments. Furthermore, there appears to be very few additional studies that specifically address unwanted negative effects of the use of VR as a therapeutic tool that are not related to cybersickness (70) or exacerbation of negative side effects with treating PTSD (71). While Albani et al (69) and our current study have some limitations, these studies can be used to alert the scientific community about the possibility of unwanted negative effects that can be resulted when using VR immersive reality for some participants.

The study's results also imply that because research within for VR is still in its infancy, there are no set procedures and/or stimuli that are primarily effective when reducing negative mood as our study concludes. Additionally, the use of VR in the medical field is relatively novel and the experiences might need to be tailored to the individual. For example, children may respond better to more game based online experiences whereas adults show preference toward natural and relaxing environments (72). Additionally, VR stimuli has also ranged in time ranging from 5 min to 60 min (73) to reduce anxiety and/or depression. These varied results further demonstrate the need to validate the most effective time of the VR experiences along with other variables in order to produce the most effective experience.

### Limitations

There are several possible limitations in this study that should be considered when examining the results. This study is unblinded and lacks a concurrently randomized control group and the participants were not completely randomly assigned to one of the six experimental conditions as those who owned a VR device were automatically placed into a VR condition and, therefore, is at risk of bias even though participants were evenly randomly assigned to one the six groups. Additionally, we were not able to control the environment atmosphere, the current condition of the VR headset, or know if there were any internet connectivity issues. While these may have been possible problems, almost all participants (97%) of participants said the experience was either “extremely clear” or “somewhat clear.” Moreover, only self-reported measurements were analyzed and thus, future studies should explore physiological variables (such as heart rate) to provide a more comprehensive evaluation of distress (74) and provide potential measurement bias online.

Due to ethical concerns of potentially transmitting Covid-19, the researchers were constrained in our ability to have participants be very active in the environment other than moving their heads around to various visuals in their experience as there were already many instructions that the participants had to follow on their own without direct help from the researchers themselves. While this approach contradicts some past research that indicated that people who can be more engaged and interact with their environment get better health results (75, 76), health standards and feasibility of increasing interaction would potentially cause other problems. Further research that can be conducted in-person would allow for there to be more interaction and engagement with what participants could do with the visuals and audio.

Another limitation is that this trial analyzed only short-term effects. Measurements were taken only at two time points (pre-intervention and post-intervention) and each patient participated in only one session. While length of traditional therapy administered by clinicians varies upon the individual, several studies indicate that in-person sessions happen over longer time and multiple sessions (77, 78) for positive psychotherapy outcomes. However, longer periods of VR intervention could lose efficacy over several sessions since individuals might habituate to it and become ineffective (i.e., VR might become less effective for increasing the positive self-esteem for people). This does remain to be assessed since a past study concluded that the benefits of VR intervention were not lost across at least three sessions, thus indicating that novelty may not play a significant role in VR enhancing well-being (79).

Furthermore, given that our sample were people that we cannot confirm nor diagnose with major depression, the generalizability of our results are limited to non-depressive adults. In other words, while our results highlight the potential positive outcomes for mood concerning depression, the results cannot firmly indicate if we'd find the same result with a population diagnosed with depression. Lastly, it should be noted that a post-hoc power analysis found the study to be statistically underpowered. Given the number of conditions and analyses, interpretations of modality effects, while in line with previous research, should be interpreted with caution. Furthermore, with the study being underpowered, there may have been some effects that were undetected.

### Future research

This study aimed to provide a modest contribution to an emerging field of study – immersive media with music for enhanced well-being. In doing so, we hope to have yielded novel insights into the effectiveness of music when delivered through VR. To our knowledge, this was a novel study in that it specifically examined the differences between 2D and VR online experiences with music to determine effectiveness of enhancing well-being. Taking into account the theoretical implications and limitations, future research should explore online environments by finding solutions to (1) shorten instructions for the setup, (2) include videos to guide the participants more clearly pictures and/or (3) have participants join online with the researcher on a video conferencing platform (e.g., Zoom or Skype) to help them with the setup of the experience and more fully control elements that were not available in this study. Additionally, researchers could explore with focus groups visual stimuli beforehand to reduce potential photosensitive images that could possibly make the environments not as effective. Furthermore, these results may indicate that 2D online environment sessions could be just as beneficial as VR but further research needs to be conducted bearing in mind the limitations and psychological considerations discussed earlier. Future research should not only explore repeated exposures for online environments but also specifically compare 2D, VR, and in-person groups to understand what is the most effective.

## Conclusion

The results of our study suggest that while both VR and 2D environments can be useful tools for improving mood for people, 2D environments were assessed as the most beneficial treatments for impacting people's psychological-well-being over a short amount of time. Sensory richness played a significant role in making 2D audiovisual the most beneficial experience for this study, but this result may be the result of psychological limitations to the VR groups. In summary, this study proposes that e-health developers and researchers should not only focus on using audiovisuals in their research but also focusing on making the onboarding process easier and exploring what visual stimuli would be most effective for enhancing well-being to meet the global clinical need for non-pharmaceutical methods. This study should provide a template for future researchers and clinicians who want to use VR and other online environments for therapeutic results and/or enhancing well-being for people.

## Data Availability

The raw data supporting the conclusions of this article will be made available by the authors, without undue reservation.
